# Phthalate Exposure, PPARα Variants, and Neurocognitive Development of Children at Two Years

**DOI:** 10.3389/fgene.2022.855544

**Published:** 2022-04-06

**Authors:** Ling Yu, Hongling Zhang, Tongzhang Zheng, Juan Liu, Xingjie Fang, Shuting Cao, Wei Xia, Shunqing Xu, Yuanyuan Li

**Affiliations:** ^1^ Key Laboratory of Environment and Health, Ministry of Education and Ministry of Environmental Protection, State Key Laboratory of Environmental Health, School of Public Health, Tongji Medical College, Huazhong University of Science and Technology, Wuhan, China; ^2^ School of Health and Nursing, Wuchang University of Technology, Wuhan, China; ^3^ Department of Epidemiology, Brown University, Providence, RI, United States

**Keywords:** phthalate metabolites, child neurodevelopment, PPARα, genetic variants, gene-environment interaction

## Abstract

**Background:** The PPARα gene may be crucial to the neurotoxic effect of phthalates. However, epidemiological studies considering the neurodevelopmental influence of phthalates interacting with genetic susceptibility are limited. We hypothesized phthalates could interact with the PPARα gene, synergistically affecting neurocognitive development.

**Methods:** A total of 961 mother-infant pairs were involved in this study. The concentrations of phthalate metabolites in maternal urine during pregnancy were detected. Children’s neurocognitive development was estimated with the Bailey Infant Development Inventory (BSID). Genetic variations in PPARα were genotyped with the Illumina Asian Screening Array. We applied generalized linear regression models to estimate genotypes and phthalate metabolites’ association with children’s neurocognitive development.

**Results:** After adjusting for potential confounders, the mono-n-butyl phthalate (MnBP) concentration was negatively associated with Psychomotor Development Index (PDI) (*β* = −0.86, 95% CI: −1.67, −0.04). The associations between MnBP and neurocognitive development might be modified by PPARα rs1800246. Compared with low-MnBP individuals carrying rs1800246 GG genotypes, high-MnBP individuals with the AG + AA genotype had a higher risk of neurocognitive developmental delay, with the odds ratio of 2.76 (95% CI:1.14, 6.24).

**Conclusions:** Our current study revealed that prenatal exposure to MnBP was negatively correlated with children’s neurocognitive development, and PPARα rs1800246 might modify the association.

## Introduction

As a group of synthetic chemical plasticizers, phthalates are extensively used in many industrial products, such as toys, stationery, packaging materials, and even cosmetics (Lyche et al., 2009). Phthalates can be slowly released from different industrial materials and accumulated in different environments, including the atmosphere, water, sediments, and soil. Therefore, the pathways of human exposure to phthalates are diverse, including gastrointestinal ingestion, respiratory inhalation, and dermal absorption (Heudorf et al., 2007).

Exposure to phthalates is a considerable threat to health worldwide (Heudorf et al., 2007; Lyche et al., 2009; Zhang et al., 2009; Ejaredar et al., 2015; Olesen et al., 2018). Developing fetuses are likely susceptible to environmental exposure owing to the rapid speed of fetal organ formation and development (Wilcox, 2010). Prenatal exposure to phthalates is neurotoxic to offspring, which has been indicated by increasing animal experiments (Miodovnik et al., 2014; Barakat et al., 2018; Hatcher et al., 2019). Recently, increasing epidemiological research explored the correlation between phthalate exposure during pregnancy and neurocognitive development in infants. However, the findings were inconsistent (Braun et al., 2014; Ejaredar et al., 2015; Bornehag et al., 2018; Engel et al., 2018; Olesen et al., 2018). Although the specific molecular mechanism of prenatal phthalate exposure affecting neurocognitive development is poorly understood, gene-environment interactions in the complex process of brain development may partly explain the inconsistency (Lai et al., 2014). It is widely acknowledged that neurodevelopment is influenced by complex interactions between genes and the environment (Lauby et al., 2021). Plastic product chemicals (PPC) were found to induce epigenetic change, which may lead to adverse offspring neurodevelopment (Ponsonby et al., 2016). Perinatal BPA exposure impaired memory in mice by inhibiting the NMDAR subunits expression (Xu et al., 2010; Xu et al., 2011). Nevertheless, studies considering genetic factors when exploring the association between phthalate exposure and neurocognitive development are limited.

Peroxisome proliferator-activated receptor α (PPARα) is a group of nuclear receptors, which plays a crucial part in adjusting fatty acid distribution and metabolism (Han et al., 2017). Prior studies have shown that phthalates can interfere with the PPARα (Hurst and Waxman, 2003; Lampen et al., 2003), which was considered part of phthalates’ neurotoxic mechanism (Miodovnik et al., 2014). PPARα has been proven to be activated *in vitro* by several phthalate diesters and monoesters (Hurst and Waxman, 2003). Besides, PPARα has also been suggested to be involved in phthalates’ developmental and reproductive toxicity (Peters et al., 1997; Ward et al., 1998; Hayashi et al., 2011; Wang et al., 2017). Therefore, we hypothesized that variants in the PPARα gene interact with phthalate exposure to influence neurocognitive development.

In the present study, we focused on evaluating the correlations between prenatal phthalate exposure and children’s neurocognitive development and exploring whether the phthalate exposure and the PPARα genetic polymorphism have interaction effects on the relationship therein based on a birth cohort study.

## Material and Methods

### Study Participants

Participants in this study were drawn from a prospective cohort study in Wuhan, China. The criteria for enrollment of pregnant women were as follows: 1) carrying singleton; 2) Wuhan residents; 3) Gestational weeks less than 16; 4) willing to give birth in the research hospital. The research protocol was approved by the ethics committees of the Tongji Medical College, Huazhong University of Science and Technology, and the Wuhan Maternal and Child Healthcare Hospital. Between October 2013 and March 2015, a total of 1,656 women donated urine samples during the first trimesters (13.0 ± 1.2 weeks) and completed the determination of urine phthalate metabolites. Then, 1,105 children accomplished the Bayley Scales of Infant Development (BSID) tests. Finally, 114 children were excluded because of the lack of results of genotyping measurement, leaving 961 mother-infant pairs for the study.

### Data Collection

Basic demographic characteristics (e.g., maternal age, education, and household income) and lifestyle factors (e.g., folic acid supplement and passive smoking during pregnancy) were obtained through face-to-face interviews using standardized and structured questionnaires. The pre-pregnancy body mass index (BMI) was calculated according to pre-pregnancy weight and height obtained from the hospital records. The information about mothers’ pregnancy history and the data of newborns (including gestational weight gain, gestational age, birth weight, and gender) were abstracted from the medical records. The gestational weight gain (GWG) (kg) was obtained by subtracting pre-pregnancy body weight (kg) from the weight (kg) at delivery. According to the Institute of Medicine and National Research Council (IOM) of the United States, the recommended total GWGs for underweight (<18.5 kg/m^2^), normal-weight (18.5–24.9 kg/m^2^), overweight (25.0–29.9 kg/m^2^), and obese (≥30.0 kg/m^2^) women are 12.5–18, 11.5–16, 7–11.5, and 5–9 kg, respectively (Medicine IO, 2009). The GWGs below this recommended range are identified as inadequate total GWG, within this recommended range are identified as adequate total GWG, and beyond this recommended range are identified as excessive total GWG.

### Measurement of Neurocognitive Development

The neurocognitive development of children was tested by three certified pediatricians using the Bayley Scales of Infant Development of China Revision (BSID-CR) in the study hospital. All study testers were unaware of the exposure information. The processes of tests were preserved via video recording. Quality control was performed through reviewing video evaluations. Two neurocognitive development indices were generated, the mental development index (MDI) and the psychomotor development index (PDI). After normalization, the mean is 100, and the standard deviation is 15. The neurocognitive developmental delay was defined as one SD or more below the mean of the scores (MDI< 85 or PDI <85) of the children in this study.

### Detection of Phthalate Metabolites

During the first trimester of pregnancy, spot urine samples were collected in 5 ml polypropylene tubes and saved at −20°C until detection. The analysis methods and instruments for phthalate metabolite measurements were described previously (Li et al., 2019). Briefly, the urine sample was hydrolyzed overnight at 37°C after adding *ß*-glucuronidase. Liquid-liquid extraction was applied three times. Then following instrumental analysis was conducted on an Ultimate 3000 UHPLC system (Dionex, Sunnyvale, CA, United States). Eight phthalate metabolites were measured including monoethyl phthalate (MEP), mono-(2-ethyl-5-carboxypentyl) phthalate (MECPP), mono-(2-ethyl-5-hydroxyhexyl) phthalate (MEHHP), mono-(2-ethyl-5-oxohexyl) phthalate (MEOHP), mono-isobutyl phthalate (MiBP), mono-n-butyl phthalate (MnBP), mono-benzyl phthalate (MBzP), and mono-(2-ethylhexyl) phthalate (MEHP). ∑DEHP was calculated as the sum of molar concentrations of DEHP metabolites (MEHP, MEHHP, MEOHP, and MECPP). ∑DBP was calculated as the sum of molar concentrations of DBP metabolites (MnBP and MiBP). The sum of the molar concentrations of MEP, MnBP, and MiBP is numerically equal to ∑LMW. Besides, the sum of the molar concentrations of MBzP, MEHP, MEHHP, MEOHP, and MECPP is numerically equal to ∑HMW.

For further analysis, we replaced values lower than the limit of detection (LOD) with values equal to half LOD. The urinary dilution was corrected as the following formula: Pc = P [(SG_m_ − 1)/(SG − 1)], where Pc means the SG-adjusted concentration (ng/ml), P means the measured concentration (ng/ml), SG is the specific gravity of sample and SG_m_ means the median SG level.

### DNA Extraction, Genotyping, and Single Nucleotide Polymorphism (SNP) Selection

Umbilical cord blood was collected immediately after delivery and transferred to vacuum blood vessels coated with EDTA. The blood samples were then centrifuged and instantly saved at −80°C until analysis. According to the standard scheme, total DNA was extracted from umbilical cord leukocytes using Wizard^®^ Genomic DNA Purification (Promega Corporation, Madison, WI, United States). According to the manufacturer’s protocol, DNA samples were genotyped using the Illumina Infinium Asian Screening Array v1.0 BeadChip (Illumina Inc., San Diego, CA, United States). The single nucleotide polymorphisms (SNPs) in PPARα (chr22:46546429-46639652, GRCh37/hg19 by Entrez Gene) were filtered out according to the criteria as follows: 1) genotype call rate <95%; 2) Hardy-Weinberg equilibrium (HWE)-P < 1 × 10^−6^; 3) minor allele count < 1Hardy-Weinberg equilibrium (HWE)-P < 1 × 10^−6^. Then imputation was performed on Michigan Imputation Server (Das et al., 2016). The reference panel was the 1,000 Genomes Project phase 3. After imputation, SNP that did not meet the following requirements was excluded: 1) minor allele frequency (MAF) > 0.05; 2) imputation quality score *R* ^2^ > 0.3. Finally, 61 SNPs in the PPARα gene were viable for further analysis.

### Statistical Analysis

The SG-adjusted phthalate metabolite levels were ln-transformed due to the skewed distribution. Descriptive statistics were conducted to describe the distributions of phthalate metabolite concentrations, neurocognitive measures, and participant characteristics. Spearman correlation coefficients were applied to summarize pairwise correlations among phthalate metabolites.

The general linear model was used to evaluate the relationships of phthalate metabolites’ concentrations with MDI or PDI. According to the previous literature (Bai et al., 2020), the coefficient *β* means that each 1% change in urinary concentrations of phthalate metabolite leads to a *β*% difference in PDI or MDI. We modeled phthalate metabolites data as quartiles (Q1-Q4) to explore the potential non-linear association. The lowest quartile (Q1) was used as the reference to estimated percent differences between quartiles. Linear trends across quartiles were calculated by modeling the median values as continuous variables. A ″2-step” approach was applied in the following gene-environment interaction analyses (Gauderman et al., 2017). Step 1: the dominant, recessive, and additive models were used to explore the associations between SNPs and neurocognitive development in the general linear models with potential confounders adjusted. Step 2: the SNPs significantly related to PDI or MDI were selected to analyze their interaction with phthalate metabolites on neurocognitive developmental delay. In order to generate hypotheses for future research, *p*-values in both steps were relaxed to 0.05. Adjustment factors were selected if they altered the main effect estimates by more than 10% or related to phthalates and children’s neurocognitive ability in the previous literature (Wehby and Murray, 2008; Téllez-Rojo et al., 2013; Botton et al., 2016). A directed acyclic graph depicts the selection of potential confounders (Supplementary Figure S1). The final models included maternal age (<25, 25-34, ≥34 years) and education (< high school degree or ≥ high school degree), gestational weight gain (inadequate, adequate, and excessive), passive smoking, and folic acid supplement during pregnancy (yes or no), parity (primiparous or multiparous), gestational age (<37 or ≥37 weeks), child gender (boys or girls), and birth weight (≤2,500, 2,500-3,999, ≥4,000 g).

All statistical analyses were performed using R, version 4.1.0. The statistical significance level was 0.05 for a two-tailed test.

## Results

### The General Characteristics of Participants

As shown in Table 1, the mean maternal age (±SD) was 29.03 ± 3.33 years. Most of the participating women were primiparous (87.30%), and approximately 66.29% of them had a pre-pregnancy BMI in the range of 18.5–23.9 kg/m^2^. The majority of the women (80.75%) had an educational background as high school or above, reported no exposure to passive smoking (66.49%), and took folic acid supplements (81.48%). The mean gestational age (±SD) was 39.34 ± 1.25 weeks. Among the children, boys accounted for 52.44%. Most children had a birth weight in the range of 2500–3999 g (93.1%). The mean MDI and PDI (±SD) for two-year-old children were 103.72 ± 22.91 and 103.77 ± 18.76, respectively.

**TABLE 1:** General characteristics of the participants (n = 961).

Characteristics	Mean ± SD or n (%)
Mothers	
Age (years)	29.03 ± 3.33
<25	77 (8.01)
25-34	804 (83.66)
≥34	80 (8.32)
Education	
≤ high school	185 (19.25)
> high school	776 (80.75)
Pre-pregnancy BMI (kg/m2)	20.79 ± 2.76
≤18.5	196 (20.40)
18.5–23.9	637 (66.29)
≥24	128 (13.32)
GWG categories by IOM recommendation	
Inadequate total GWG	135 (14.04)
Adequate total GWG	369 (38.40)
Excessive total GWG	457 (47.55)
Passive smoking during pregnancy	
No	639 (66.49)
Yes	322 (33.51)
Folic acid supplement during pregnancy	
No	178 (18.52)
Yes	783 (81.48)
Parity	
Primiparous	839 (87.30)
Multiparous	122 (12.70)
Children	
Gestational age (weeks)	39.34 ± 1.25
<37	34 (3.54)
≥37	927 (96.46)
Gender	
Male	504 (52.44)
Female	457 (47.55)
Birth weight (g)	3,297.54 ± 413.82
≤2,500	27 (2.81)
2,500-3,999	894 (93.03)
≥4,000	40 (4.16)
MDI scores	103.72 ± 22.91
<85	169 (17.59)
≥85	792 (82.41)
PDI scores	103.77 ± 18.76
<85	137 (14.26)
≥85	824 (85.74)

Abbreviations: SD, standard deviation; BMI, body mass index; GWG, gestational weight gain; IOM, International Organization of Medicine; MDI, mental development index; PDI, psychomotor development index.

Note: According to the IOM, the recommended total GWGs, for underweight (<18.5 kg/m2), normal-weight (18.5–24.9 kg/m2), overweight (25.0–29.9 kg/m2), and obese (≥30.0 kg/m2) women are 12.5–18, 11.5–16, 7–11.5, and 5–9 kg, respectively. The GWGs, below this recommended range are identified as inadequate total GWG, within this recommended range are identified as adequate total GWG, and beyond this recommended range are identified as excessive total GWG.

### Concentrations of Urinary Phthalate Metabolites

The distribution of urinary phthalate metabolite concentrations is shown in Table 2. All eight phthalate metabolites were detected in at least 84.9% of urine samples. MnBP had the highest concentration (geometric mean = 54.28 ng/ml), while the lowest concentration of phthalate metabolites was MBzP (geometric mean = 0.09 ng/ml). The concentration of ∑DEHP was equal to ∑HMW same as 0.09 nmol/ml, considerably lower than ∑DBP (0.36 nmol/ml) and ∑LMW (0.47 nmol/ml). Metabolite pairwise correlation coefficients varied from 0.19 to 1.00 (Supplementary Table S2).

**TABLE 2:** Distributions of urinary phthalate metabolite concentrations (ng/ml).

Metabolites	LOD	DF%	Percentile			GM (SD)
			25th	50th	75th	
MEP	0.10	100.0	5.37	10.20	22.02	10.50 (3.94)
MECPP	0.01	100.0	6.44	9.82	16.99	9.89 (3.01)
MEHHP	0.05	100.0	4.49	7.26	12.32	7.14 (2.81)
MEOHP	0.05	100.0	3.23	5.20	8.55	5.10 (2.75)
MiBP	0.10	100.0	11.59	20.09	36.23	16.98 (4.19)
MnBP	0.10	100.0	28.25	56.63	121.87	54.28 (4.27)
MBzP	0.10	84.9	0.04	0.09	0.23	0.09 (4.05)
MEHP	0.10	100.0	2.06	4.48	8.29	3.67 (4.16)
∑DEHP	NA	NA	0.07	0.10	0.16	0.09 (2.60)
∑DBP	NA	NA	0.21	0.37	0.72	0.36 (3.71)
∑LMW	NA	NA	0.27	0.47	0.91	0.47 (3.24)
∑HMW	NA	NA	0.06	0.10	0.16	0.09 (2.59)

Abbreviations: LOD, limit of detection; DF, detection frequency; GM, geometric mean; SD, standard deviation; MEP, mono-ethyl phthalate; MECPP, mono-(2-ethyl-5-carboxypentyl) phthalate; MEHHP, mono-(2-ethyl-5-hydroxyhexyl) phthalate; MEOHP, mono-(2-ethyl-5-oxohexyl) phthalate; MiBP- mono-isobutyl phthalate; MnBP, mono-n-butyl phthalate; MBzP - monobenzyl phthalate; MEHP, mono-2-ethylhexyl phthalate; ∑DEHP, sum of di(2-ethylhexyl) phthalate metabolites. ∑DEHP, represent the sum of molar concentrations (nmol/ml) of MEHP, MEHHP, MEOHP, and MECPP; ∑DBP, represent the sum of molar concentrations (nmol/ml) of MnBP, and MiBP; ∑LMW, represent the sum of molar concentrations (nmol/ml) of MEP, MnBP, and MiBP; ∑HMW, represent the sum of molar concentrations (nmol/ml) of MBzP, MEHP, MEHHP, MEOHP, and MECPP.

### Associations Between Urinary Phthalate Metabolites and Neurocognitive Development

After adjusting for potential confounders, MnBP was found to be negatively associated with PDI (Table 3). The regression coefficients in Table 3 indicated that PDI averagely decreased 0.86% (95% CI: −1.67, −0.04) with a 1% increase in urinary concentration of MnBP. We found no significant association between MDI and phthalate metabolites. Although on-linear associations were explored, we did not find significant non-linear associations (Supplementary Table S3). Thus, MnBP was selected to analyze their interaction with PPARα variants.

**TABLE 3:** Associations between phthalate metabolite concentrations and neurocognitive development.

Phthalate Metabolites (ng/ml)	MDI	PDI
	β (95%CIs)	β (95%CIs)
MEP	0.48 (−0.56, 1.51)	−0.13 (−0.99, 0.74)
MECPP	−0.53 (−1.81, 0.76)	−0.16 (−1.23, 0.92)
MEHHP	0.15 (−1.21, 1.52)	−0.20 (−1.34, 0.94)
MEOHP	−0.04 (−1.44, 1.36)	−0.09 (−1.26, 1.08)
MiBP	−0.00 (−0.98, 0.99)	−0.69 (−1.52, 0.13)
**MnBP**	−0.01 (−0.99, 0.97)	−**0.86 (**−**1.67,** −**0.04)**
MBzP	0.49 (−0.52, 1.51)	0.50 (−0.34, 1.35)
MEHP	−0.14 (−1.14, 0.86)	0.03 (−0.81, 0.86)
∑DEHP	−0.61 (−3.40, 2.19)	−0.53 (−2.87, 1.80)
∑DBW	0.38 (−0.16, 0.93)	−0.16 (−0.61, 0.30)
∑LMW	0.42 (−0.12, 0.96)	−0.15 (−0.60, 0.31)
∑HMW	−0.59 (−3.38, 2.21)	−0.52 (−2.85, 1.82)

Note: The general linear model was specified in the “Material and methods” section. Bold numbers indicated that the association was significant. The unit of ∑DEHP ∑DBP ∑LMW, and ∑HMW, was nmol/mL. The coefficient *ß* indicated that each 1% change in urinary concentrations of phthalate metabolite leads to a *β*% difference in PDI, or MDI.Adjustment: Maternal age, maternal education, gestational weight gain, passive smoking during pregnancy, folic acid supplement during pregnancy, parity, gestational age, child gender, and birth weight.

### Associations of PPARα Variants With Neurocognitive Development

In Table 4, it was shown that rs75525202-G and rs1800246-A were negatively associated with MDI in both additive and dominant manners (*P*_*add*_ = 0.027, 0.004, and *P*_*dom*_ = 0.032, 0.007). While, rs4823902-G, rs4253690-G and rs5766698-T were positively associated with MDI in both additive and dominant manners (*P*_*add*_ = 0.033, 0.042, 0.028, and *P*_*dom*_ = 0.010,0.022, 0.007). The rs12330015-G and rs115250492-T were positively related to MDI in dominant manner (rs12330015 GA + GG vs AA genotype, *β* = 3.291, *P*_*dom*_ = 0.040; rs115250492 TA + TT vs AA genotype, *β* = 3.453, *P*_*dom*_ = 0.041).

**TABLE 4:** Associations of the seven selected PPARα variants with neurocognitive development.

SNP	Major/Minor	Genotyped/Imputed	MAF	MDI						PDI					
				Additive Model		Dominant Model		Recessive Model		Additive Model		Dominant Model		Recessive Model	
				β	*P*_*add*_	β	*P*_*dom*_	β	*P*_*rec*_	β	*P*_*add*_	β	*P*_*dom*_	β	*P*_*rec*_
rs75525202	A/G	Genotyped	0.116	−**3.601**	**0.027**	−**3.732**	**0.032**	−6.736	0.373	−0.830	0.544	−0.512	0.725	−8.103	0.199
rs4823902	T/G	Genotyped	0.207	**2.651**	**0.033**	**3.869**	**0.010**	0.038	0.991	0.999	0.338	1.321	0.295	0.710	0.802
rs4253690	A/G	Genotyped	0.164	2.788	0.042	**3.602**	**0.022**	0.770	0.858	0.630	0.583	0.983	0.457	−1.080	0.764
rs12330015	A/G	Genotyped	0.154	2.372	0.092	**3.291**	**0.040**	−1.771	0.703	0.235	0.842	0.615	0.647	−2.583	0.506
rs5766698	T/C	Imputed	0.193	**2.772**	**0.028**	**4.105**	**0.007**	−0.177	0.959	0.617	0.559	0.778	0.544	0.655	0.821
rs115250492	A/T	Imputed	0.132	2.475	0.092	**3.453**	**0.041**	−1.299	0.784	0.176	0.886	0.323	0.820	−0.700	0.861
rs1800246	G/A	Genotyped	0.070	−**5.837**	**0.004**	−**5.681**	**0.007**	−24.45	0.123	−2.847	0.097	−2.740	0.118	−13.500	0.308

Abbreviation: Major, Major allele; Minor, Minor allele; MAF, minor allele frequency; dom, dominant; rec, recessive; add, additive.

Notes: The general linear model was specified in the “Material and methods” section. Bold numbers indicated that the association was significant. The dominant, recessive, and additive models were conducted to investigate the associations between variants and Bayley scores with adjustment for maternal age, maternal education, gestational weight gain, passive smoking during pregnancy, folic acid supplement during pregnancy, gestational age, parity, child gender, and infant birth weight. Reference subgroups were wild homozygote and wild homozygote + heterozygote for dominant and recessive models, respectively. In the additive model, *p* values were calculated for the trend of *β* with the increased number of minor alleles.

These seven SNPs were selected to further explore their interaction with MnBP exposure because they were the most likely to be related to neurocognitive development among the PPARα variants.

### Effects of Interaction Between MnBP and SNPs in PPARα Gene

Table 5 presents the interaction between urinary MnBP concentration and seven candidate SNPs on neurocognitive developmental delay.

**TABLE 5:** Interaction between urinary MnBP and seven selected PPARα variants on neurocognitive developmental delay.

SNP	N	MDI Domain			PDI Domain		
		n (%)	OR (95%CI)	Interaction OR (95% CI)	n (%)	OR (95%CI)	Interaction OR (95% CI)
rs75525202							
MnBP_L/AA	556	102 (18.35%)	Reference	1.87 (0.72, 4.75)	41 (7.37%)	Reference	1.03 (0.38, 2.68)
MnBP_L/GA + GG	164	32 (19.51%)	1.08 (0.67, 1.69)		56 (34.15%)	1.13 (0.67, 1.86)	
MnBP_H/AA	192	24 (12.50%)	0.65 (0.39, 1.04)		22 (11.46%)	1.27 (0.79, 2.01)	
MnBP_H/GA + GG	49	11 (22.45%)	1.3 (0.60, 2.64)		18 (36.73%)	1.48 (0.64, 3.11)	
rs4823902							
MnBP_L/TT	451	91 (20.18%)	Reference	1.02 (0.40, 2.49)	70 (15.52%)	Reference	1.34 (0.54, 3.24)
MnBP_L/GT + GG	269	43 (15.99%)	0.74 (0.49, 1.11)		27 (10.04%)	**0.59 (0.36, 0.95)**	
MnBP_H/TT	159	25 (15.72%)	0.74 (0.44, 1.22)		28 (17.61%)	1.16 (0.70, 1.88)	
MnBP_H/GT + GG	82	10 (12.20%)	0.56 (0.26, 1.10)		12 (14.63%)	0.92 (0.45, 1.76)	
rs4253690							
MnBP_L/AA	507	98 (19.33%)	Reference	0.73 (0.27, 1.86)	75 (14.79%)	Reference	0.93 (0.35, 2.40)
MnBP_L/GA + GG	213	36 (16.90%)	0.84 (0.54, 1.29)		22 (10.33%)	0.64 (0.38, 1.05)	
MnBP_H/AA	167	27 (16.17%)	0.82 (0.50, 1.33)		31 (18.56%)	1.31 (0.81, 2.09)	
MnBP_H/GA + GG	74	8 (10.81%)	0.51 (0.22, 1.05)		9 (12.16%)	0.78 (0.35, 1.58)	
rs12330015							
MnBP_L/AA	515	102 (19.81%)	Reference	0.91 (0.33, 2.37)	54 (10.49%)	Reference	0.76 (0.27, 1.97)
MnBP_L/GA + GG	205	32 (15.61%)	0.74 (0.47, 1.15)		43 (20.98%)	0.78 (0.47, 1.27)	
MnBP_H/AA	174	27 (15.52%)	0.77 (0.47, 1.24)		28 (16.09%)	1.36 (0.84, 2.17)	
MnBP_H/GA + GG	67	8 (11.94%)	0.52 (0.22, 1.09)		12 (17.91%)	0.81 (0.34, 1.68)	
rs5766698							
MnBP_L/TT	472	95 (20.13%)	Reference	0.87 (0.33, 2.18)	67 (14.19%)	-	0.56 (0.21, 1.43)
MnBP_L/CT + CC	248	39 (15.73%)	0.72 (0.47, 1.09)		30 (12.10%)	0.82 (0.51, 1.3)	
MnBP_H/TT	163	26 (15.95%)	0.78 (0.47, 1.27)		32 (19.63%)	1.48 (0.91, 2.37)	
MnBP_H/CT + CC	78	9 (11.54%)	0.49 (0.22, 0.99)		6 (7.69%)	0.68 (0.29, 1.42)	
rs115250492							
MnBP_L/AA	548	106 (19.34%)	Reference	0.71 (0.23, 1.97)	75 (13.69%)	Reference	0.41 (0.12, 1.19)
MnBP_L/TA + TT	172	28 (16.28%)	0.8 (0.49, 1.26)		22 (12.79%)	0.92 (0.54, 1.53)	
MnBP_H/AA	182	29 (15.93%)	0.81 (0.50, 1.29)		35 (19.23%)	1.52 (0.96, 2.38)	
MnBP_H/TA + TT	59	6 (10.17%)	0.46 (0.17, 1.04)		5 (8.47%)	0.57 (0.19, 1.37)	
rs1800246							
MnBP_L/GG	616	107 (17.37%)	Reference	2.14 (0.71, 6.20)	41 (6.66%)	Reference	**3.56 (1.16, 10.85)**
MnBP_L/AG + AA	104	27 (25.96%)	1.55 (0.92, 2.55)		56 (53.85%)	0.74 (0.37, 1.37)	
MnBP_H/GG	212	27 (12.74%)	0.68 (0.42, 1.07)		20 (9.43%)	1.05 (0.66, 1.64)	
MnBP_H/AG + AA	29	8 (27.59%)	2.25 (0.88, 5.27)		20 (68.97%)	**2.76 (1.14, 6.24)**	

Notes: The general linear model was specified in the “Material and methods” section. Bold numbers indicated that the association was significant. MnBP_L urinary MnBP, concentration ≤75th percentile; MnBP_H urinary MnBP, concentration >75th percentile; MDI, domain, MDI <85; PDI, domain, PDI <85. All models were adjusted for maternal age, maternal education, gestational weight gain, passive smoking during pregnancy, folic acid supplement during pregnancy, gestational age, parity, child gender, and infant birth weight. *P*_*interaction*_ was calculated by introducing a multiplicative interaction term (dichotomous urinary MnBP × genotypes) into the generalized linear models.

For the PDI domain, compared to subjects with low urinary MnBP (≤121.873 ng/ml) and carrying rs1800246 GG genotype, the subjects with high urinary MnBP (>121.873 ng/ml) and carrying rs1800246AG + AA genotype had a 2.76-fold risk for neurocognitive developmental delay (95% CI: 1.14,6.24). Modification effects on the relationship between urinary MnBP and neurocognitive developmental delay were found in rs1800246. Compared to subjects with low urinary MnBP and carrying rs4823902 TT genotype, the subjects with high urinary MnBP and rs4823902 GT + GG genotype had a 0.59-fold risk for neurocognitive developmental delay (95% CI: 0.36,0.95). However, rs4823902 was not found to modify the association between urinary MnBP and PDI. As for the MDI domain, no potential interaction between MnBP and selected SNPs in the PPARα gene was found.

## Discussion

In this study, we investigated the associations of prenatal phthalate exposure with neurocognitive development in 2-year-old children. We found that MnBP in maternal urinary were negatively related to PDI, and genetic variations in the PPARα gene might have interaction effects with high MnBP on the neurocognitive developmental delay in children.

We tested urinary phthalate levels and found that phthalate metabolites were detectable in all study participants, suggesting that humans are extensively exposed to phthalates. Prior studies have also indicated that phthalates are ubiquitous in the environment because they are widely used in industrial products (Heudorf et al., 2007; Wittassek and Angerer, 2008; Miodovnik et al., 2014). Given the extensive adverse effects of phthalate on health, molecular studies are desperately needed to provide mechanistic insight into the health effects of phthalate.

A negative association was also found between prenatal urinary MnBP concentration and PDI, consistent with the previous studies (Kim et al., 2011; Polanska et al., 2014; Qian et al., 2019). A study conducted in New York City revealed the inverse association between MnBP concentrations in maternal urine samples during pregnancy and psychomotor development in 296 children (Whyatt et al., 2012). Another study also observed that concentrations (μg/g creatinine) of 3OH-MnBP were inversely associated with motor development in 150 children (Polanska et al., 2014). In animal experiments, DBP, the precursor of MnBP, has been shown to impair neurocognitive development in mice. Li et al. (2009) reported that high maternal exposure to DBP lead to delayed surface righting and shortened forepaw grip time in rat pups. Additionally, Lee et al. (2020) reported that perinatal DBP exposure significantly reduced the motor and memory abilities of puppies after delivery. Nevertheless, the specific mechanisms linking prenatal phthalate exposures to neurocognitive development remain unclear. A recent study reported that DBP might induce neurotoxicity in human neurons by disrupting the expression of estrogen receptors (Xu et al., 2020). In addition, interference with thyroid homeostasis, calcium signaling, lipid metabolism, and peroxisome proliferator-activated receptor (PPAR) activation may also be part of the neurotoxic mechanism (Miodovnik et al., 2014).

Neurocognitive development is a complex process accompanied by long-term interactions between genetic and environmental factors. Although phthalates may have adverse effects on development through multiple different molecular targets, there is considerable evidence that the toxicity of phthalates is partly driven by the effect of PPARα. (Hurst and Waxman, 2003; Lampen et al., 2003; Bility et al., 2004; Corton and Lapinskas, 2005; Lapinskas et al., 2005; Kawano et al., 2014). PPARα is a critical receptor expressed in the human brain tissue and has extensive developmental effects (Abbott, 2009). Previous studies indicated that DBP was capable of activating PPARα in cells (Mandard et al., 2004; Corton and Lapinskas, 2005; Froment, 2008; Latini et al., 2008; Lau et al., 2010; Hayashi et al., 2011). Experiments on animals also demonstrated that hepatic PPARα was vital to the toxic effect of perinatal phthalate exposure in the offspring (Hayashi et al., 2011). In order to generate hypotheses for future research, *p*-values in both steps of the 2-step gene-by-environment approach were relaxed to 0.05 for a total alpha of 0.10. Although this 2-step approach did not yield any clear gene-by-environment interaction SNPs, seven PPARα variants were found to be associated with performance on the mental development index. Additionally, for one of these, rs1800246, there was evidence of potential interaction between urinary MnBP concentration on psychomotor index performance. The functional role of rs1800246 is not well documented in the literature. Nonetheless, the SNP functional annotations tool HaploReg v4.1 revealed that rs1800246, an intronic SNP, may affect the expression of the PPARα gene through cis-acting regulatory elements that positively control gene expressions, such as enhancers, silencers, insulators, and transcription factors. (Deng et al., 2017).

Although the mechanism is unclear, the study provides clues to the interaction between PPARα and phthalates in developing neurocognitive functions. Further research is required to investigate the potential interaction between environmental and genetic factors on children’s neurocognitive development.

Several advantages exist in the present research. Firstly, this study explored the association between phthalate exposure and PPARα gene polymorphisms with neurocognitive development based on a prospective cohort study. Since exposures were assessed before outcomes occurred, they were less biased. As far as we know, this is one of the few studies examining the interaction of urinary phthalate and genetic polymorphisms on neurocognitive development, which confirms the importance of environment-gene interactions on neurocognitive development and provides clues for the prevention of neurocognitive developmental delay. Nevertheless, some limitations in this study should be noticed. Firstly, the lack of correction for multiple testing and the cross-over of outcomes increased the chance of a false positive finding. Therefore, independently replicated cohort studies or *in vitro* experiments are required to verify the results and explore the potential mechanisms. Finally, although we had adjusted many confounders in this study, there may still be other potential or uncontrolled factors that affect the results.

## Conclusion

Our present study provides evidence that prenatal phthalate exposure is related to worse neurocognitive development in children, which may be modified by PPARα polymorphisms. The results offered epidemiological evidence about the interaction between prenatal phthalate exposure and inherited factors on neurocognitive development in children and provided clues for preventing neurocognitive developmental delay in the early stage.

## Data Availability

The datasets generated and used during the current study are not publicly available due to the potential for individual and organizational privacy to be compromised. Requests to access the datasets should be directed to the corresponding author.
