# Supplementary material for: Phthalate Exposure, PPARα Variants, and Neurocognitive Development of Children at Two Years
Source: Front Genet. 2022 Apr 6;13:855544. doi: 10.3389/fgene.2022.855544 (PMC9019295; doi:10.3389/fgene.2022.855544)
Supplement: Supplementary file 1 [file Table1.DOCX]

| **Table S1. Comparison of the basic characteristics of the analyzed population and the excluded population** | | | | |  |
| --- | --- | --- | --- | --- | --- |
| Characteristics | Analysis population (n = 961) |  | Excluded population (n = 695) | *P*-Value^a^ | |
|  | Mean ± SD or n (%) |  | Mean ± SD or n (%) |  |  |
| Mothers |  |  |  |  | |
| Age (years) | 29.03 ± 3.33 |  | 28.46 ± 3.45 | 0.32 | |
| <25 | 77 (8.01) |  | 61 (8.77) |  | |
| 25-34 | 804 (83.66) |  | 589 (84.78) |  | |
| ≥34 | 80 (8.32) |  | 45 (6.45) |  | |
| Education |  |  |  | 0.16 | |
| ≤ high school | 185 (19.25) |  | 131 (18.85) |  | |
| > high school | 776 (80.75) |  | 564 (81.12) |  | |
| Pre-pregnancy BMI (kg/m2) | 20.79 ± 2.76 |  | 20.45 ± 2.39 | 0.58 | |
| ≤18.5 | 196 (20.40) |  | 152 (21.88) |  | |
| 18.5-23.9 | 637 (66.29) |  | 468 (67.34) |  | |
| ≥24 | 128 (13.32) |  | 75 (10.79) |  | |
| GWG categories by IOM recommendation | |  |  | 0.34 | |
| Inadequate total GWG | 135 (14.04) |  | 91 (13.09) |  | |
| Adequate total GWG | 369 (38.40) |  | 279 (40.11) |  | |
| Excessive total GWG | 457 (47.55) |  | 325 (46.78) |  | |
| Passive smoking during pregnancy | |  |  | 0.23 | |
| No | 639 (66.49) |  | 470 (67.65) |  | |
| Yes | 322 (33.51) |  | 225 (32.35) |  | |
| Folic acid supplement during pregnancy | |  |  | 0.14 | |
| No | 178 (18.52) |  | 115 (16.56) |  | |
| Yes | 783 (81.48) |  | 580 (83.44) |  | |
| Parity |  |  |  | 0.58 | |
| Primiparous | 839 (87.30) |  | 601 (86.47) |  | |
| Multiparous | 122 (12.70) |  | 94 (13.53) |  | |
| Children |  |  |  |  | |
| Gestational age(weeks) | 39.34 ± 1.25 |  | 39.67 ± 1.34 | 0.76 | |
| <37 | 34 (3.54) |  | 21 (3.02) |  | |
| ≥37 | 927 (96.46) |  | 674 (96.98) |  | |
| Gender |  |  |  | 0.54 | |
| Male | 504 (52.44) |  | 505 (52.44) |  | |
| Female | 457 (47.55) |  | 458 (47.55) |  | |
| Birth weight (g) | 3297.54 ± 413.82 |  | 3297.54 ± 413.83 | 0.45 | |
| ≤2500 | 27 (2.81) |  | 15 (2.16) |  | |
| 2500-3999 | 894 (93.03) |  | 644 (92.66) |  | |
| ≥4000 | 40 (4.16) |  | 36 (5.19) |  | |
| MDI scores | 103.72 ± 22.91 |  | 103.67 ± 21.89 | 0.24 | |
| <85 | 169 (17.59) |  | 111 (15.97) |  | |
| ≥85 | 792 (82.41) |  | 584 (84.03) |  | |
| PDI scores | 103.77 ± 18.76 |  | 103.33 ± 20.72 | 0.31 | |
| <85 | 137 (14.26) |  | 109 (15.68) |  | |
| ≥85 | 824 (85.74) |  | 586 (84.32) |  | |
| Abbreviations: SD, standard deviation; BMI, body mass index; GWG, gestational weight gain; IOM, International Organization of Medicine; MDI, mental development index; PDI, psychomotor development index.  a *P*-Value for comparison between analysis population and excluded population; non-parametric tests were used for comparisons of linear variables; chi-square tests were used for comparisons of categorical variables. | | | | |  |
