# Supplementary material for: Phthalate Exposure, PPARα Variants, and Neurocognitive Development of Children at Two Years
Source: Front Genet. 2022 Apr 6;13:855544. doi: 10.3389/fgene.2022.855544 (PMC9019295; doi:10.3389/fgene.2022.855544)
Supplement: Supplementary file 2 [file Table2.DOCX]

| **Table S2. Pairwise relationships between specific gravity-adjusted urinary phthalate metabolites. Spearman correlation coefficients are shown.** | | | | | | | | | | | | |
| --- | --- | --- | --- | --- | --- | --- | --- | --- | --- | --- | --- | --- |
|  | MEP | MECPP | MEHHP | MEOHP | MiBP | MnBP | MBzP | MEHP | ∑DEHP | ∑DBW | ∑LMW | ∑HMW |
| MEP | 1.00 |  |  |  |  |  |  |  |  |  |  |  |
| MECPP | 0.25 | 1.00 |  |  |  |  |  |  |  |  |  |  |
| MEHHP | 0.20 | **0.89** | 1.00 |  |  |  |  |  |  |  |  |  |
| MEOHP | 0.22 | **0.92** | **0.96** | 1.00 |  |  |  |  |  |  |  |  |
| MiBP | 0.28 | 0.30 | 0.33 | 0.35 | 1.00 |  |  |  |  |  |  |  |
| MnBP | 0.27 | 0.34 | 0.39 | 0.40 | 0.55 | 1.00 |  |  |  |  |  |  |
| MBzP | 0.23 | 0.30 | 0.35 | 0.36 | 0.32 | 0.38 | 1.00 |  |  |  |  |  |
| MEHP | 0.20 | 0.52 | 0.59 | 0.58 | 0.23 | 0.21 | 0.19 | 1.00 |  |  |  |  |
| ∑DEHP | 0.23 | **0.89** | **0.92** | **0.91** | 0.33 | 0.38 | 0.31 | **0.76** | 1.00 |  |  |  |
| ∑DBW | 0.30 | 0.35 | 0.40 | 0.41 | **0.72** | **0.96** | 0.39 | 0.23 | 0.39 | 1.00 |  |  |
| ∑LMW | 0.52 | 0.37 | 0.39 | 0.41 | 0.68 | **0.90** | 0.40 | 0.25 | 0.40 | **0.94** | 1.00 |  |
| ∑HMW | 0.23 | **0.89** | **0.92** | **0.91** | 0.34 | 0.38 | 0.32 | 0.76 | **1.00** | 0.40 | 0.41 | 1.00 |
| Note: The bold indicated spearman correlation coefficients > 0.70. | | | | | | | | | | | | |
