# Supplementary material for: Phthalate Exposure, PPARα Variants, and Neurocognitive Development of Children at Two Years
Source: Front Genet. 2022 Apr 6;13:855544. doi: 10.3389/fgene.2022.855544 (PMC9019295; doi:10.3389/fgene.2022.855544)
Supplement: Supplementary file 3 [file Table3.DOCX]

| **Table S3. Associations between quartile-categorized urinary phthalate metabolites and neurocognitive development.** | | | | | |
| --- | --- | --- | --- | --- | --- |
| Phthalate metabolites | MDI | |  | PDI | |
|  | β (95%CI) | *P_trend_* |  | β (95%CI) | *P_trend_* |
| MEP |  |  |  |  |  |
| Q1 (<5.37 ng/mL) | Reference | 0.379 |  | Reference | 0.960 |
| Q2 (5.37 ~ 10.20 ng/mL) | 2.10 (-1.91, 6.10) |  |  | 1.39 (-1.95, 4.74) |  |
| Q3 (10.20 ~ 22.02 ng/mL) | 3.43 (-0.59, 7.45) |  |  | 2.57 (-0.79, 5.94) |  |
| Q4 (>22.02 ng/mL) | 2.61 (-1.41, 6.63) |  |  | 0.81 (-2.55, 4.17) |  |
| MECPP |  |  |  |  |  |
| Q1 (<6.44 ng/mL) | Reference | 0.195 |  | Reference | 0.428 |
| Q2 (6.44 ~ 9.82 ng/mL) | 2.37 (-1.63, 6.38) |  |  | 3.22 (-0.12, 6.56) |  |
| Q3 (9.82 ~ 16.99 ng/mL) | 1.61 (-2.42, 5.63) |  |  | 1.31 (-2.06, 4.67) |  |
| Q4 (>16.99 ng/mL) | 3.18 (-0.83, 7.18) |  |  | 2.35 (-0.99, 5.69) |  |
| MEHHP |  |  |  |  |  |
| Q1 (<4.49 ng/mL) | Reference | 0.057 |  | Reference | 0.371 |
| Q2 (4.49 ~ 7.26 ng/mL) | 1.26 (-2.74, 5.26) |  |  | 1.235 (-2.11, 4.581) |  |
| Q3 (7.26 ~ 12.32 ng/mL) | 2.75 (-1.25, 6.76) |  |  | 2.75 (-0.599, 6.098) |  |
| Q4 (>12.32 ng/mL) | 3.87 (-0.13, 7.87) |  |  | 1.786 (-1.56, 5.132) |  |
| MEOHP |  |  |  |  |  |
| Q1 (<3.23 ng/mL) | Reference | 0.052 |  | Reference | 0.363 |
| Q2 (3.23 ~ 5.20 ng/mL) | 1.75 (-2.25, 5.75) |  |  | -0.65 (-3.99, 2.69) |  |
| Q3 (5.20 ~ 8.55 ng/mL) | 0.55 (-3.47, 4.56) |  |  | 1.53 (-1.83, 4.88) |  |
| Q4 (>8.55 ng/mL) | **4.08 (0.08, 8.08)** |  |  | 1.16 (-2.19, 4.51) |  |
| MiBP |  |  |  |  |  |
| Q1 (<11.59 ng/mL) | Reference | 0.930 |  | Reference | 0.053 |
| Q2 (11.59 ~ 20.09 ng/mL) | 1.51 (-2.51, 5.53) |  |  | -0.67 (-4.02, 2.67) |  |
| Q3 (20.09 ~ 36.23 ng/mL) | -0.88 (-5.15, 3.38) |  |  | **-4.10 (-7.65, -0.54)** |  |
| Q4 (>36.23 ng/mL) | 0.66 (-3.18, 4.49) |  |  | -3.32 (-6.51, -0.12) |  |
| MnBP |  |  |  |  |  |
| Q1 (<28.25 ng/mL) | Reference | 0.113 |  | Reference | 0.778 |
| Q2 (28.25 ~ 56.63 ng/mL) | -3.70 (-7.69, 0.29) |  |  | -2.69 (-6.04, 0.65) |  |
| Q3 (56.63 ~ 121.87 ng/mL) | -0.60 (-4.61, 3.40) |  |  | -0.82 (-4.18, 2.54) |  |
| Q4 (>121.87 ng/mL) | 1.35 (-2.64, 5.34) |  |  | -1.46 (-4.80, 1.88) |  |
| MBzP |  |  |  |  |  |
| Q1 (<0.04 ng/mL) | Reference | 0.077 |  | Reference | 0.315 |
| Q2 (0.04 ~ 0.09 ng/mL) | -3.48 (-7.48, 0.52) |  |  | -0.43 (-3.79, 2.92) |  |
| Q3 (0.09 ~ 0.23 ng/mL) | 2.24 (-1.76, 6.23) |  |  | 2.44 (-0.91, 5.78) |  |
| Q4 (>0.23 ng/mL) | 1.88 (-2.10, 5.87) |  |  | 1.46 (-1.89, 4.80) |  |
| MEHP |  |  |  |  |  |
| Q1 (<2.06 ng/mL) | Reference | 0.724 |  | Reference | 0.926 |
| Q2 (2.06 ~ 4.48 ng/mL) | 0.12 (-3.88, 4.13) |  |  | 0.72 (-2.64, 4.07) |  |
| Q3 (4.48 ~ 8.29 ng/mL) | 1.47 (-2.54, 5.48) |  |  | 0.70 (-2.66, 4.05) |  |
| Q4 (>8.29 ng/mL) | 0.68 (-3.35, 4.69) |  |  | 0.39 (-2.97, 3.75) |  |
| ∑DEHP |  |  |  |  |  |
| Q1 (<0.07 nmol/mL) | Reference | 0.320 |  | Reference | 0.775 |
| Q2 (0.07 ~ 0.10 nmol/mL) | 1.32 (-2.69, 5.32) |  |  | -0.31 (-3.66, 3.04) |  |
| Q3 (0.10 ~ 0.16 nmol/mL) | 0.49 (-3.52, 4.50) |  |  | 1.63 (-1.73, 4.98) |  |
| Q4 (>0.16 nmol/mL) | 2.26 (-1.76, 6.27) |  |  | -0.56 (-3.91, 2.79) |  |
| ∑DBW |  |  |  |  |  |
| Q1 (<0.21 nmol/mL) | Reference | 0.224 |  | Reference | 0.807 |
| Q2 (0.21 ~ 0.37 nmol/mL) | -3.68 (-7.67, 0.32) |  |  | -3.12 (-6.46, 0.23) |  |
| Q3 (0.37 ~ 0.72 nmol/mL) | 1.04 (-2.98, 5.05) |  |  | -1.33 (-4.70, 2.03) |  |
| Q4 (>0.72 nmol/mL) | 0.84 (-3.15, 4.83) |  |  | -1.57 (-4.92, 1.77) |  |
| ∑LMW |  |  |  |  |  |
| Q1 (<0.27 nmol/mL) | Reference | 0.099 |  | Reference | 0.817 |
| Q2 (0.27 ~ 0.47 nmol/mL) | -2.40 (-6.43, 1.63) |  |  | **-4.87 (-8.23, -1.510)** |  |
| Q3 (0.47 ~ 0.91 nmol/mL) | 0.88 (-3.16, 4.91) |  |  | -0.95 (-4.32, 2.42) |  |
| Q4 (>0.91 nmol/mL) | 2.12 (-1.88, 6.11) |  |  | -2.05 (-5.38, 1.28) |  |
| ∑HMW |  |  |  |  |  |
| Q1 (<0.06 nmol/mL) | Reference | 0.280 |  | Reference | 0.814 |
| Q2 (0.06 ~ 0.10 nmol/mL) | 1.05 (-2.96, 5.05) |  |  | -0.40 (-3.75, 2.95) |  |
| Q3 (0.10 ~ 0.16 nmol/mL) | -0.09 (-4.10, 3.93) |  |  | 1.23 (-2.13, 4.59) |  |
| Q4 (>0.16 nmol/mL) | 2.29 (-1.71, 6.30) |  |  | -0.45 (-3.80, 2.90) |  |
| Note: The general linear model was specified in the “Material and methods” section. Bold numbers indicated that the association was significant. All models were adjusted for maternal age, maternal education, gestational weight gain, passive smoking during pregnancy, folic acid supplement during pregnancy, gestational age, parity, child gender, and infant birth weight. | | | | | |
