# Supplementary material for: Phthalate Exposure, PPARα Variants, and Neurocognitive Development of Children at Two Years
Source: Front Genet. 2022 Apr 6;13:855544. doi: 10.3389/fgene.2022.855544 (PMC9019295; doi:10.3389/fgene.2022.855544)
Supplement: Supplementary file 4 [file Table4.DOCX]

| **Table S4. The associations between 61 PPARα variants and** **neurocognitive development.** | | | | | | | | | | | | | | | | | |
| --- | --- | --- | --- | --- | --- | --- | --- | --- | --- | --- | --- | --- | --- | --- | --- | --- | --- |
| SNPs | Location | Major /Minor | MAF | R^2^ | Genotyped  /Imputed | **MDI** | | | | | | **PDI** | | | | | |
|  |  |  |  |  |  | Additive model | | Dominant model | | Recessive model | | Additive model | | Dominant model | | Recessive model | |
|  |  |  |  |  |  | β | *P_add_* | β | *P_dom_* | β | *P_rec_* | β | *P_add_* | β | *P_dom_* | β | *P_rec_* |
| rs5767320 | 22:46544914 | T/A | 0.146 | 0.916 | Imputed | 0.699 | 0.626 | 0.826 | 0.612 | 0.672 | 0.887 | 1.768 | 0.140 | 2.053 | 0.132 | 2.007 | 0.613 |
| rs4253623 | 22:46550106 | A/G | 0.146 | 0.915 | Imputed | 0.706 | 0.621 | 0.815 | 0.617 | 0.880 | 0.849 | 1.670 | 0.162 | 1.921 | 0.159 | 2.143 | 0.581 |
| rs135555 | 22:46550315 | G/C | 0.069 | 0.413 | Imputed | -0.907 | 0.646 | -1.633 | 0.446 | 8.950 | 0.292 | 0.015 | 0.992 | 0.182 | 0.919 | -2.586 | 0.716 |
| rs5769112 | 22:46550916 | A/C | 0.133 | 0.717 | Imputed | 0.713 | 0.629 | 0.882 | 0.601 | 0.403 | 0.933 | 1.710 | 0.166 | 2.050 | 0.146 | 1.518 | 0.708 |
| rs135552 | 22:46552815 | T/C | 0.071 | 0.416 | Imputed | -0.494 | 0.799 | -1.282 | 0.547 | 9.631 | 0.225 | -0.066 | 0.967 | 0.031 | 0.986 | -1.530 | 0.818 |
| rs135551 | 22:46553021 | G/A | 0.071 | 0.413 | Imputed | -1.167 | 0.551 | -1.919 | 0.364 | 8.950 | 0.292 | -0.012 | 0.993 | 0.146 | 0.934 | -2.586 | 0.716 |
| rs135550 | 22:46553234 | T/C | 0.071 | 0.417 | Imputed | -1.167 | 0.551 | -1.919 | 0.364 | 8.950 | 0.292 | -0.012 | 0.993 | 0.146 | 0.934 | -2.586 | 0.716 |
| rs135549 | 22:46553308 | T/C | 0.225 | 0.631 | Imputed | 0.107 | 0.928 | 0.291 | 0.844 | -0.527 | 0.862 | 1.124 | 0.260 | 1.247 | 0.314 | 2.049 | 0.420 |
| rs135548 | 22:46553503 | A/G | 0.069 | 0.418 | Imputed | -0.907 | 0.646 | -1.633 | 0.446 | 8.950 | 0.292 | 0.015 | 0.992 | 0.182 | 0.919 | -2.586 | 0.716 |
| rs135547 | 22:46553650 | C/G | 0.073 | 0.417 | Imputed | -1.160 | 0.549 | -1.892 | 0.365 | 8.950 | 0.292 | -0.149 | 0.926 | -0.017 | 0.992 | -2.586 | 0.716 |
| rs135545 | 22:46554042 | C/T | 0.073 | 0.417 | Imputed | -1.160 | 0.549 | -1.892 | 0.365 | 8.950 | 0.292 | -0.149 | 0.926 | -0.017 | 0.992 | -2.586 | 0.716 |
| rs135543 | 22:46555321 | C/T | 0.070 | 0.414 | Imputed | -0.838 | 0.670 | -1.545 | 0.468 | 8.950 | 0.292 | -0.050 | 0.975 | 0.104 | 0.953 | -2.586 | 0.716 |
| rs135542 | 22:46556037 | T/C | 0.070 | 0.417 | Imputed | -0.832 | 0.673 | -1.541 | 0.470 | 8.950 | 0.292 | 0.324 | 0.844 | 0.543 | 0.761 | -2.586 | 0.716 |
| rs135541 | 22:46556366 | G/T | 0.072 | 0.41 | Imputed | -0.906 | 0.638 | -1.758 | 0.404 | 9.631 | 0.225 | 0.308 | 0.848 | 0.474 | 0.787 | -1.530 | 0.818 |
| rs135540 | 22:46556612 | T/A | 0.072 | 0.416 | Imputed | -1.192 | 0.540 | -1.942 | 0.356 | 8.950 | 0.292 | -0.231 | 0.887 | -0.111 | 0.949 | -2.586 | 0.716 |
| rs129600 | 22:46557161 | A/G | 0.386 | 0.685 | Imputed | -0.355 | 0.736 | 0.020 | 0.989 | -1.357 | 0.504 | -0.763 | 0.386 | -0.573 | 0.647 | -1.787 | 0.293 |
| rs135539 | 22:46559267 | C/A | 0.448 | 0.976 | Genotyped | 0.015 | 0.988 | 0.854 | 0.586 | -1.068 | 0.550 | -0.172 | 0.840 | -0.303 | 0.817 | -0.134 | 0.928 |
| rs62225951 | 22:46560011 | A/G | 0.150 | 0.953 | Imputed | 0.380 | 0.788 | 0.511 | 0.752 | -0.091 | 0.983 | 1.530 | 0.195 | 1.774 | 0.190 | 1.871 | 0.623 |
| rs5769175 | 22:46560261 | A/C | 0.152 | 0.947 | Imputed | 0.141 | 0.920 | 0.196 | 0.903 | -0.091 | 0.983 | 1.395 | 0.236 | 1.594 | 0.237 | 1.871 | 0.623 |
| rs5769177 | 22:46561135 | A/G | 0.153 | 0.964 | Imputed | 0.324 | 0.817 | 0.394 | 0.807 | 0.273 | 0.951 | 1.616 | 0.168 | 1.698 | 0.208 | 3.389 | 0.364 |
| rs5769178 | 22:46561274 | A/C | 0.150 | 0.975 | Imputed | 0.531 | 0.707 | 0.710 | 0.661 | -0.091 | 0.983 | 1.609 | 0.173 | 1.878 | 0.165 | 1.871 | 0.623 |
| rs9627046 | 22:46561566 | G/A | 0.150 | 0.996 | Genotyped | 0.645 | 0.648 | 0.859 | 0.596 | -0.091 | 0.983 | 1.529 | 0.195 | 1.773 | 0.190 | 1.871 | 0.623 |
| rs76755807 | 22:46561713 | A/G | 0.150 | 0.975 | Imputed | 0.531 | 0.707 | 0.710 | 0.661 | -0.091 | 0.983 | 1.609 | 0.173 | 1.878 | 0.165 | 1.871 | 0.623 |
| rs9627047 | 22:46562173 | T/G | 0.151 | 0.982 | Imputed | 0.454 | 0.747 | 0.607 | 0.707 | -0.091 | 0.983 | 1.482 | 0.208 | 1.709 | 0.205 | 1.871 | 0.623 |
| rs9626730 | 22:46562183 | T/C | 0.151 | 0.998 | Genotyped | 0.454 | 0.747 | 0.607 | 0.707 | -0.091 | 0.983 | 1.482 | 0.208 | 1.709 | 0.205 | 1.871 | 0.623 |
| rs4253785 | 22:46564135 | T/C | 0.149 | 0.96 | Imputed | 0.653 | 0.644 | 0.872 | 0.591 | -0.091 | 0.983 | 1.737 | 0.142 | 2.051 | 0.131 | 1.871 | 0.623 |
| rs4253648 | 22:46564230 | A/G | 0.149 | 0.973 | Imputed | 0.650 | 0.646 | 0.867 | 0.593 | -0.091 | 0.983 | 1.643 | 0.164 | 1.926 | 0.155 | 1.871 | 0.623 |
| rs135538 | 22:46564628 | G/C | 0.487 | 0.715 | Imputed | -0.492 | 0.636 | 0.441 | 0.790 | -1.828 | 0.288 | -0.455 | 0.600 | -0.868 | 0.531 | -0.310 | 0.829 |
| **rs75525202** | **22:46565612** | **A/G** | **0.120** | **0.980** | **Genotyped** | **-3.601** | **0.027** | **-3.732** | **0.032** | **-6.736** | **0.372** | **-0.830** | **0.543** | **-0.512** | **0.725** | **-8.103** | **0.199** |
| rs12484245 | 22:46566330 | T/C | 0.149 | 0.970 | Imputed | 0.653 | 0.644 | 0.872 | 0.591 | -0.091 | 0.983 | 1.737 | 0.142 | 2.051 | 0.131 | 1.871 | 0.623 |
| rs881740 | 22:46567388 | A/G | 0.151 | 0.994 | Genotyped | 0.667 | 0.633 | 0.892 | 0.582 | 0.018 | 0.996 | 1.869 | 0.109 | 1.905 | 0.159 | 4.419 | 0.228 |
| rs135536 | 22:46568318 | G/C | 0.067 | 0.415 | Imputed | -1.644 | 0.422 | *-*2.032 | 0.343 | 6.174 | 0.582 | -0.329 | 0.847 | -0.227 | 0.899 | -3.686 | 0.694 |
| rs9626736 | 22:46570232 | A/G | 0.112 | 0.960 | Genotyped | -0.950 | 0.551 | -0.896 | 0.613 | -3.041 | 0.601 | 0.919 | 0.490 | 0.921 | 0.535 | 2.367 | 0.627 |
| **rs4823902** | **22:46579365** | **T/G** | **0.210** | **0.990** | **Genotyped** | **2.651** | **0.033** | **3.869** | **0.010** | **0.038** | **0.991** | **0.999** | **0.337** | **1.321** | **0.294** | **0.710** | **0.802** |
| rs4253681 | 22:46579600 | T/C | 0.207 | 0.977 | Imputed | 2.723 | 0.058 | 3.869 | 0.510 | 0.672 | 0.841 | 1.091 | 0.294 | 1.321 | 0.294 | 1.401 | 0.617 |
| **rs4253690** | **22:46580923** | **A/G** | **0.160** | **0.990** | **Genotyped** | **2.788** | **0.041** | **3.602** | **0.022** | **0.770** | **0.858** | **0.632** | **0.582** | **0.983** | **0.456** | **-1.080** | **0.763** |
| **rs12330015** | **22:46590278** | **A/G** | **0.150** | **0.990** | **Genotyped** | **2.372** | **0.092** | **3.291** | **0.040** | **-1.771** | **0.702** | **0.235** | **0.841** | **0.615** | **0.647** | **-2.583** | **0.505** |
| **rs5766698** | **22:46593236** | **T/C** | **0.190** | **0.900** | **Imputed** | **2.772** | **0.028** | **4.105** | **0.007** | **-0.177** | **0.959** | **0.617** | **0.559** | **0.778** | **0.543** | **0.655** | **0.820** |
| **rs115250492** | **22:46593818** | **A/T** | **0.130** | **0.890** | **Imputed** | **2.475** | **0.092** | **3.453** | **0.041** | **-1.299** | **0.784** | **0.176** | **0.886** | **0.323** | **0.819** | **-0.696** | **0.860** |
| rs4253709 | 22:46594750 | G/T | 0.174 | 0.792 | Imputed | 1.810 | 0.167 | 2.689 | 0.085 | -0.570 | 0.877 | 0.087 | 0.936 | 0.189 | 0.885 | -0.361 | 0.907 |
| rs5767560 | 22:46595546 | A/T | 0.131 | 0.835 | Imputed | 2.201 | 0.136 | 3.096 | 0.067 | -1.662 | 0.731 | 0.542 | 0.660 | 1.024 | 0.469 | -2.537 | 0.530 |
| rs4823613 | 22:46598307 | A/G | 0.235 | 0.980 | Genotyped | -1.015 | 0.394 | -1.555 | 0.290 | 0.051 | 0.986 | 0.903 | 0.365 | 1.035 | 0.399 | 1.460 | 0.567 |
| rs8141725 | 22:46598631 | T/C | 0.234 | 0.940 | Imputed | -0.996 | 0.405 | -1.537 | 0.295 | 0.135 | 0.965 | 0.955 | 0.339 | 1.071 | 0.383 | 1.641 | 0.524 |
| rs55688271 | 22:46604397 | A/G | 0.169 | 0.964 | Imputed | -0.337 | 0.802 | -0.485 | 0.756 | 0.189 | 0.963 | 0.041 | 0.970 | 0.040 | 0.975 | 0.113 | 0.973 |
| rs35637220 | 22:46604816 | T/C | 0.167 | 0.924 | Imputed | -0.548 | 0.685 | -0.548 | 0.685 | 0.555 | 0.893 | 0.061 | 0.957 | -0.090 | 0.945 | 1.203 | 0.729 |
| rs5767634 | 22:46604855 | G/A | 0.169 | 0.990 | Genotyped | -0.337 | 0.802 | -0.485 | 0.756 | 0.189 | 0.963 | 0.041 | 0.970 | 0.040 | 0.975 | 0.113 | 0.973 |
| rs5767636 | 22:46605104 | A/G | 0.169 | 0.952 | Imputed | -0.337 | 0.802 | -0.485 | 0.756 | 0.189 | 0.963 | 0.041 | 0.970 | 0.040 | 0.975 | 0.113 | 0.973 |
| rs5766741 | 22:46605190 | T/C | 0.169 | 0.942 | Imputed | -0.337 | 0.802 | -0.485 | 0.756 | 0.189 | 0.963 | 0.041 | 0.970 | 0.040 | 0.975 | 0.113 | 0.973 |
| rs5766743 | 22:46607378 | A/G | 0.145 | 0.798 | Imputed | 0.314 | 0.824 | 0.529 | 0.746 | -0.864 | 0.849 | 0.477 | 0.688 | 0.850 | 0.534 | -1.669 | 0.660 |
| rs4253730 | 22:46610409 | A/G | 0.187 | 0.808 | Imputed | 0.861 | 0.506 | 0.489 | 0.749 | 4.283 | 0.254 | 1.009 | 0.351 | 1.455 | 0.255 | -0.306 | 0.922 |
| rs41397552 | 22:46611263 | G/A | 0.091 | 0.992 | Genotyped | 0.571 | 0.749 | 1.037 | 0.585 | -7.854 | 0.357 | 1.223 | 0.413 | 1.468 | 0.356 | -1.678 | 0.814 |
| rs4253737 | 22:46611435 | T/G | 0.204 | 0.835 | Imputed | 0.333 | 0.791 | -0.089 | 0.952 | 3.117 | 0.377 | 0.493 | 0.639 | 0.603 | 0.630 | 0.536 | 0.856 |
| rs5767700 | 22:46612672 | T/C | 0.199 | 0.82 | Imputed | 0.304 | 0.809 | -0.133 | 0.929 | 3.117 | 0.377 | 0.670 | 0.525 | 0.859 | 0.495 | 0.536 | 0.856 |
| rs4253747 | 22:46613237 | T/A | 0.199 | 0.817 | Imputed | 0.296 | 0.814 | -0.145 | 0.923 | 3.117 | 0.377 | 0.662 | 0.530 | 0.847 | 0.501 | 0.536 | 0.856 |
| rs4253749 | 22:46613798 | G/A | 0.197 | 0.828 | Imputed | 0.267 | 0.832 | -0.188 | 0.901 | 3.117 | 0.377 | 0.730 | 0.489 | 0.946 | 0.454 | 0.536 | 0.856 |
| rs4253750 | 22:46613852 | T/C | 0.199 | 0.823 | Imputed | 0.304 | 0.809 | -0.133 | 0.929 | 3.117 | 0.377 | 0.670 | 0.525 | 0.859 | 0.495 | 0.536 | 0.856 |
| rs4253751 | 22:46613980 | A/G | 0.204 | 0.823 | Imputed | -0.107 | 0.932 | -0.658 | 0.660 | 2.747 | 0.431 | 0.757 | 0.470 | 0.942 | 0.452 | 0.762 | 0.794 |
| **rs1800246** | **22:46615625** | **G/A** | **0.070** | **0.970** | **Genotyped** | **-5.837** | **0.004** | **-5.681** | **0.006** | **-24.45** | **0.123** | **-2.847** | **0.096** | **-2.744** | **0.118** | **-13.510** | **0.308** |
| rs4253758 | 22:46616732 | T/C | 0.269 | 0.773 | Imputed | -1.203 | 0.298 | -2.248 | 0.120 | 1.359 | 0.629 | -0.050 | 0.958 | -0.463 | 0.702 | 1.453 | 0.537 |
| rs6007662 | 22:46621045 | A/G | 0.150 | 0.958 | Genotyped | 0.557 | 0.697 | 0.373 | 0.816 | 3.108 | 0.529 | 1.381 | 0.249 | 1.351 | 0.315 | 3.686 | 0.372 |
| rs5767743 | 22:46621994 | T/C | 0.177 | 0.587 | Imputed | 0.601 | 0.648 | 0.176 | 0.909 | 4.150 | 0.288 | 1.167 | 0.290 | 1.105 | 0.393 | 3.202 | 0.326 |
| **Abbreviation:** Major, Major allele; Minor, Minor allele; MAF, minor allele frequency; Major, major allele; Minor, minor allele; dom, dominant; rec, recessive; add, additive. | | | | | | | | | | | | | | | | | |
| Notes: The general linear model was specified in the “Material and methods” section. Bold numbers indicated that the association was significant. Dominant, recessive, and additive models were conducted to investigate the associations between PPARs variants and neurocognitive development in the generalized linear regression, with adjustment for maternal age, maternal education, gestational weight gain, passive smoking during pregnancy, folic acid supplement during pregnancy, gestational age, parity, child gender, and infant birth weight. In the additive model, *P* values were calculated for the trend of β with an increased number of minor alleles. The bold indicated a significant association between SNPs and neurocognitive development. | | | | | | | | | | | | | | | | | |
