# Supplementary figures and images for: Phthalate Exposure, PPARα Variants, and Neurocognitive Development of Children at Two Years
Source: Front Genet. 2022 Apr 6;13:855544. doi: 10.3389/fgene.2022.855544 (PMC9019295; doi:10.3389/fgene.2022.855544)

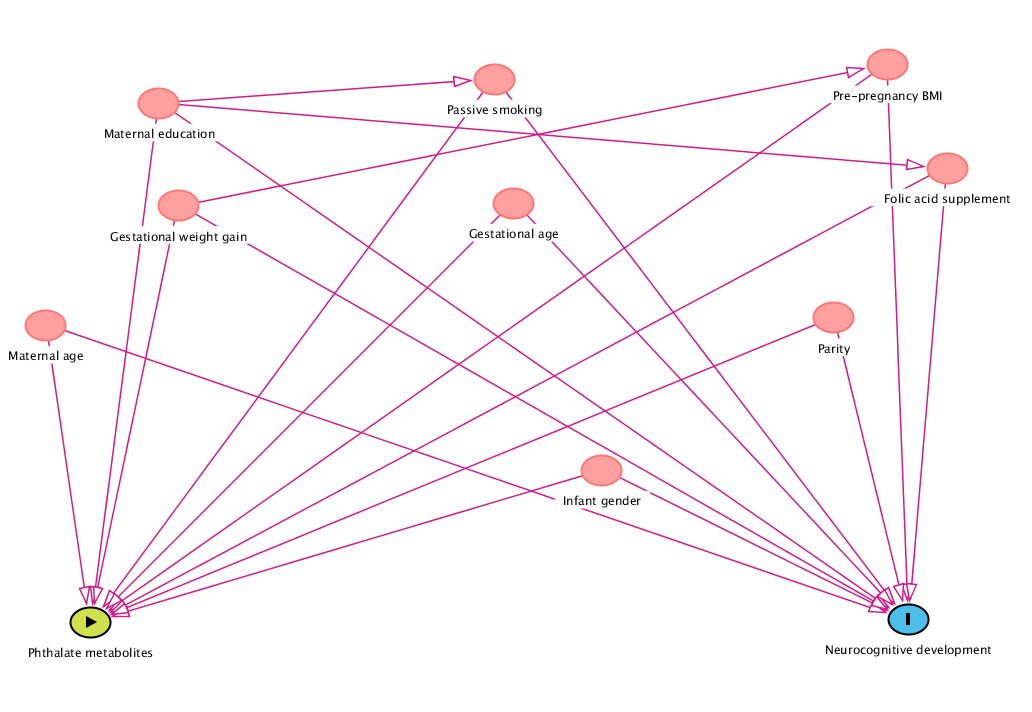

Supplement: Supplementary file 5 [file Image1.PNG]
